# *Wolbachia *infections that reduce immature insect survival: Predicted impacts on population replacement

**DOI:** 10.1186/1471-2148-11-290

**Published:** 2011-10-05

**Authors:** Philip R Crain, James W Mains, Eunho Suh, Yunxin Huang, Philip H Crowley, Stephen L Dobson

**Affiliations:** 1 Department of Entomology, College of Agriculture, University of Kentucky, Lexington, KY 40546-0091, USA; 2 Department of Biology, Center for Ecology, Evolution and Behavior, University of Kentucky, Lexington, KY 40502-0225, USA

## Abstract

**Background:**

The evolutionary success of *Wolbachia *bacteria, infections of which are widespread in invertebrates, is largely attributed to an ability to manipulate host reproduction without imposing substantial fitness costs. Here, we describe a stage-structured model with deterministic immature lifestages and a stochastic adult female lifestage. Simulations were conducted to better understand *Wolbachia *invasions into uninfected host populations. The model includes conventional *Wolbachia *parameters (the level of cytoplasmic incompatibility, maternal inheritance, the relative fecundity of infected females, and the initial *Wolbachia *infection frequency) and a new parameter termed relative larval viability (*RLV*), which is the survival of infected larvae relative to uninfected larvae.

**Results:**

The results predict the *RLV *parameter to be the most important determinant for *Wolbachia *invasion and establishment. Specifically, the fitness of infected immature hosts must be close to equal to that of uninfected hosts before population replacement can occur. Furthermore, minute decreases in *RLV *inhibit the invasion of *Wolbachia *despite high levels of cytoplasmic incompatibility, maternal inheritance, and low adult fitness costs.

**Conclusions:**

The model described here takes a novel approach to understanding the spread of *Wolbachia *through a population with explicit dynamics. By combining a stochastic female adult lifestage and deterministic immature/adult male lifestages, the model predicts that even those *Wolbachia *infections that cause minor decreases in immature survival are unlikely to invade and spread within the host population. The results are discussed in relation to recent theoretical and empirical studies of natural population replacement events and proposed applied research, which would use *Wolbachia *as a tool to manipulate insect populations.

## Background

**Figure 1:**
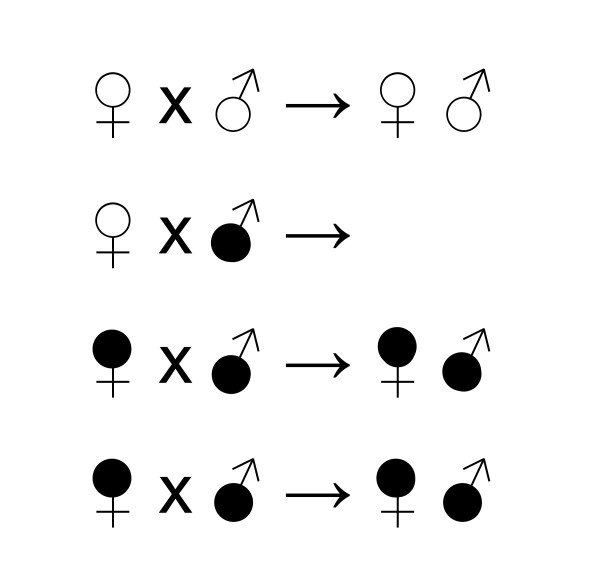
**Unidirectional cytoplasmic incompatibility crossing pattern**. White circles represent uninfected individuals and black circles represent *Wolbachia *infected individuals. Crosses between the same infection type produces viable offspring. Cytoplasmic incompatibility occurs when uninfected females mate with *Wolbachia *infected males, resulting in reduced numbers of viable offspring. As a result, infected females have an effective mating advantage over uninfected females.

The success of obligate endosymbiotic organisms depends on their ability to invade, establish and persist in their host. *Wolbachia pipientis*, a well-studied endosymbiont, is a species of maternally inherited bacteria in the order Rickettsiales, and infections are estimated to occur in more than half of all insect species [1]. Prior studies have demonstrated the ability of *Wolbachia *to manipulate the reproduction of its host [2,3]; several phenotypes have been described, including male-killing [4,5], feminization [6,7], parthenogenesis [8-10], and cytoplasmic incompatibility (CI) [11-13]. CI affects a broad range of insect taxa and causes a reduction in egg hatch when *Wolbachia-*uninfected females and *Wolbachia*-infected males mate (Figure 1).

Prior models highlight three *Wolbachia-*specific parameters that affect the probability of *Wolbachia *invasion and establishment: the maternal inheritance rate, which is the proportion of infected offspring produced by an infected female; the level of CI, which is the proportion of embryos that fail to develop as a result of incompatible crosses [14]; and the fitness cost to females for carrying a *Wolbachia *infection, defined as a decrease in overall fecundity [15-20].

Previous studies predict that the successful invasion of *Wolbachia *into an uninfected host population requires low fecundity costs, high maternal inheritance rates, and high levels of CI [21,22]. *Wolbachia *infections that impose a 10% relative fecundity cost to adult females experience reductions in their invasion success [21]. Similarly, low maternal inheritance reduces the probability of *Wolbachia *invasion [22]. Higher initial *Wolbachia *infection frequencies are predicted to increase the probability of population replacement, which can offset the above costs [14]. Models have also addressed population structure at the adult stage, impacts on adult survival, stochastic effects, and overlapping generations [14,21-25].

The relative importance of *Wolbachia *effects on immature life stages has not been assessed theoretically. This is despite multiple examples demonstrating an effect of *Wolbachia *on immature hosts. In the stored product pest *Liposcelis tricolor *(Psocoptera: Liposcelidae), *Wolbachia *infections can decrease development periods and increase survivorship in some immature life stages [26]. Other studies demonstrate negative impacts of *Wolbachia *infections on larval survival and development time [27,28]. Recent studies have determined that when intraspecific competition is intense, *Wolbachia*-infected mosquito larvae experience reduced survival [29,30].

To better understand population replacement by CI-inducing *Wolbachia*, we have evaluated both *Wolbachia *infection dynamics and host population dynamics using a model that includes deterministic immature and adult male lifestages and a stochastic adult female lifestage. Since *Wolbachia *are transmitted maternally, the sex and infection status of hosts are explicit, and adult females are tracked individually. The focus of this modeling approach was to investigate changes in the probability of population replacement resulting from varying the relative larval viability (*RLV*), expressed as relative survival of infected to uninfected larvae. The results are presented in context with traditional parameters: the rate of CI, maternal inheritance (*MI*), the relative fecundity of infected females (*RF*), and the initial *Wolbachia *infection frequency (*IF*), on the probability of population replacement.

## Methods

The model simulates a panmictic population that is closed to immigrants and emigrants. Consistent with previous studies, the model assumes mating is random and that *Wolbachia *infection has no effect on mating success. Females in the model mate once immediately upon reaching maturity. Adult survival is density-independent, but larval survival is density-dependent. The model presented here combines a stochastic adult female stage with deterministic adult male and immature stages. By implementing a deterministic immature stage, additional information regarding population dynamics is incorporated without developing a completely stochastic model, which would be considerably more computationally-intensive. The model incorporates overlapping generations [24] while tracking major life stages and considers females and males separately. Development time and survival during immature stages are addressed explicitly by the model. The model was designed assuming the host is a holometabolous insect, and the model was parameterized based upon estimates of mosquitoes in the genus *Aedes *as a case study.

### Brief Description of Equations

The following is a brief overview of all equations and parameters implemented in the model presented here. Additional development details, initial parameter values, and sensitivity analysis are provided in Additional File 1.

(1) $$R=\frac{\left(j-h\right)\Delta te^{-qB}+h\Delta t}{s}$$

Larval development rate *R *(developmental stage units): *j *is the maximum development rate (developmental stage units), *h *is the asymptotic minimum development rate (developmental stage units), *Δt *is the time step (units of time), *q *is the density-dependent development coefficient (units of (mass)^-1^), *B *is the total larval biomass (units of mass) and *s *is the total number of developmental stages. Derived from Gavotte *et al. *[30] and comparable to previously published data [31,32].

(2) $$S_{L}=e^{-\left(\mu +\alpha B^{\beta }+\gamma d^{-\varepsilon }\right)\Delta t}$$

Larval survival, *S*_*L*_: μ is the baseline mortality rate of mosquito larvae in the absence of competition (units of (time)^-1^). *α *is the coefficient controlling density dependent mortality (units of (time)^-1^). *B *is the total larval biomass (dimensionless), *β *is the exponent controlling density dependent mortality (dimensionless), *γ *is the coefficient that decreases mortality as development stage increases (units of (time)^-1^), *d *is the developmental stage index, *ε *is the exponent that decreases mortality as development stage increases (dimensionless), and *Δt *which is the time step (units of time). Based on Dye [33] and similar to previously published studies [34-36].

(3) $$M=\frac{m_{x}e^{k\left(d-1\right)}}{1+{\frac{1-c}{c}}^{\frac{T_{o}-T}{T_{O}}}}$$

Mosquito body mass, *M *(units of mass): *m*_*x *_(units of mass) is the theoretical maximum mass of a given mosquito at time *T*. *m*_*x *_is linked to *c *(dimensionless), which is the percent of *m*_*x *_that is attainable. *k *(dimensionless) is the growth coefficient; *T*_*0 *_(dimensionless) is the development time at which mass at pupation is *m*_*x*_/2 days, and *T *(dimensionless) is development time. *d *(dimensionless) represents the total number of development stages completed by the larval cohort. Derived from previously published data [30].

(4) $$F_{s}=e^{-gA}$$

Female survivorship, *F*_*s*_: *g *is the per capita mortality rate of adult females (units of (time)^-1^) and *A *is the current age of the female (units of time). Taken from Trpis and Hausermann [37].

(5) $$E=u\Delta te^{v{\left(M_{f}+w\right)}^{z}}$$

Egg production, *E*: *u *is the egg production rate; *Δt *is the time step (units of time); *v *is the female mass coefficient (units of (mass)^-1^); *M*_*f *_is the body mass of the ovipositing female (units of mass); *w *is the female mass intercept (units of mass), and *z *is the female mass exponent (dimensionless). Derived by combining two previously published functions [38,39].

### Immature Life Stages

**Figure 2:**
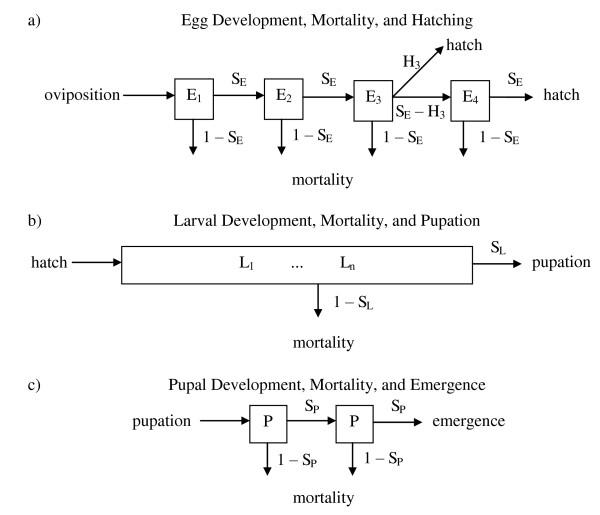
**Immature population structure**. a) Eggs develop through four discrete stages and each stage is one day. There are two cohorts of eggs, *Wolbachia *uninfected and infected. During development, eggs move through each stage consecutively, and the number of eggs advancing to the next stage reflects the product of the number of eggs present and *S*_*E*_, daily egg survivorship (Table S1). All eggs hatch after four days except a proportion of eggs hatch at day three (*H*_*3*_, Table S1). b) Larvae develop through *s *discrete stages, where *s *is an arbitrary number of developmental stages (*s *= 30). Larvae are divided into four categories: *Wolbachia *infected/uninfected and male/female. Larvae move *R *developmental stages in each time step, where *R *is the number of developmental stages a larval cohort will progress (Equation 1). The number of larvae progressing from their current development stage, e.g. *L*_*2*_, to their next developmental stage, *L*_*2+R*_, is equal to the product of the number of larvae in a developmental stage and larval survival (Equation 2). Larval survival and development are density dependent. If larvae are *Wolbachia *infected, they are subject also to the parameter *RLV *(Table 1), which can reduce the number of surviving larvae. Larvae that reach the last developmental stage become pupae. c) Pupae progress through two discrete development stages and are tracked similar to eggs. Each pupal developmental stage is one day and pupae are subject to *S*_*P*_, daily pupal survivorship (Table S1).

To simulate variation in egg hatch, the model assumes that some eggs (proportion equal to *H*_*3*_, Table S1 in Additional file 1) hatch on day three while the remaining eggs (1-*H*_*3*_) hatch on day four (Figure 2a) [40,41]. Eggs are separated into two cohorts based on infection status. Larvae are distributed into four categories for each of the possible combinations of sex and infection status.

Larvae develop through discrete developmental stages, where the development rate is affected by density dependence, and larval survival is subject to both stage-dependent mortality and density-dependence (Figure 2b). The term "stage" is defined here as a measure of progress through larval development. The number of these discrete developmental stages is chosen to allow for variation in development time and is otherwise arbitrary (i.e., not linked to age or developmental instar explicitly). The number of larval developmental stages, *s*, can be varied, but was set to *s *= 30 for this study. Larval development rate, *R*, is the number of developmental stages through which a cohort of larvae will pass within 24 hours (Equation 1). The number of larvae surviving to the next day is the product of the number of larvae in the preceding time period and the larval survival rate (Equation 2). When the number of developmental stages within a day is not an integer, the larval cohort is distributed into two adjacent developmental stages in proportions that preserve the average development rate. The latter also introduces variation into the development rates of larval cohorts (Figure 2b). Density-dependence is based on the total mass of larvae (Equation 3). Male and female cohorts are considered separately to observe sex-specific patterns during development. For example, female mosquitoes require longer development time to become adults relative to males, and studies demonstrate that males and females respond to competition intensities differently [30].

Uninfected larval cohorts progress through development subject to stage-dependent mortality and density dependent effects only. Infected larval cohorts are subject also to a reduction in viability associated with *Wolbachia *infection. The relative larval viability (*RLV*, Table 1) for infected larvae is a proportion that indicates the relative survival of infected to uninfected larvae.

**Table 1:** Glossary of notation, including the initial values for each key parameter

symbol	definition	initial value
*CI*	proportion of embryos not hatching in incompatible CI crosses	0.999
*MI*	proportion of offspring receiving infection (maternal inheritance)	0.999
*RF*	relative fecundity of infected females to uninfected females	0.999
*RLV*	relative larval viability of infected larvae to uninfected larvae	0.999
*IF*	initial frequency of gravid infected females to the total adult population	0.500

In all subsequent model runs, each value remains constant while one key parameter is varied. (For a list of all population dynamic parameters, see Table S1 in Additional file 1.)

Following the completion of larval development stages, individuals become non-feeding pupae, which have a daily survival that is independent of population density (*S*_*p*_, Table S1; Figure 2c). After completing pupal development, emerging male adults are tracked separately as either infected or uninfected cohorts. Emerging female adults are tracked as individuals.

### Adult Life Stages

Six variables are tracked over time and determine the state of individual females: the blood meal state (time since last feeding), age (days since emerging), *Wolbachia *infection status (infected or uninfected), the *Wolbachia *infection status of her mate (determined randomly based on the proportion of infected males in the population at the time she mates), size (body mass), and reproductive state (the number of gonotrophic cycles completed).

The probability that a female obtains a blood meal is determined by the frequency of potential blood meals per unit area, and each blood meal is associated with an additional mortality risk, regardless of mosquito age (Table S1). In the panmictic population simulated here, the availability of potential blood meals is assumed to be constant, but the model will allow downstream population structuring and geographic variation of bloodmeal availability.

Adult female daily survivorship *F*_*s *_is age-dependent and probabilistic (Equation 4) [37]. A female that is *Wolbachia *uninfected and mated with an infected male will lay eggs, but a proportion of the eggs will not hatch, depending on the level of CI (Table 1). Infected females produce viable offspring regardless of their mate's infection status but are subject to a decrease in relative fecundity (*RF*, Table 1). The number of eggs laid by an individual female is determined by her mass (Equation 5), and larval development influences female body mass. Specifically, intense competition delays development and reduces the mass of adult females.

Adult males, which are dead end hosts for *Wolbachia*, are not tracked individually but are tracked as infected and uninfected cohorts. The male mortality rate is assumed to be age-independent and constant (*S*_*M*_, Table S1). The proportion of *Wolbachia *infected males in the population determines the probability of an incompatible mating for uninfected females.

### Simulations

**Figure 3:**
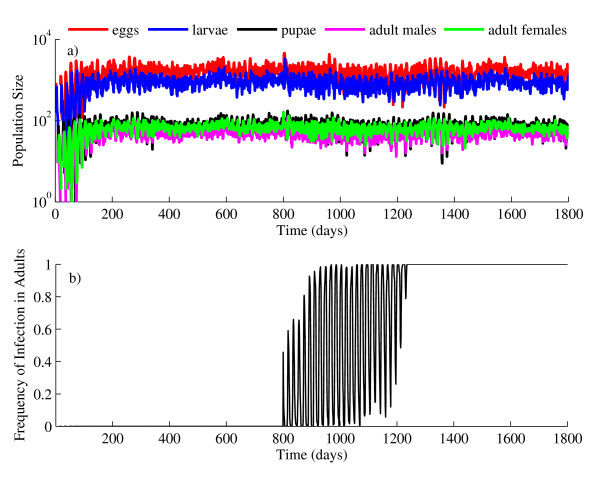
**Example of typical population dynamics produced by a simulation of the model**. a) Populations begin with an uninfected cohort of eggs. The population is allowed to persist and self-regulate for 800 days, at which time *Wolbachia *is introduced to the population as gravid, bloodfed females at the rate defined in Table 1. The population is then allowed to self-regulate and persist until 1800 days have elapsed. b) The proportion of the female population that is infected with *Wolbachia *over time (i.e., infection frequency), demonstrating a population replacement event.

The model was written in MATLAB 7 (The MathWorks Inc., Natick, MA). A single simulation of the model produced population dynamics that are tracked over time (Figure 3). A series of simulations (n = 1000) were used to assess the impact of incremental parameter changes on the probability of population replacement. The parameters emphasized were cytoplasmic incompatibility (*CI*); maternal inheritance (*MI*); the relative fecundity of adult females (*RF*); the initial *Wolbachia *infection frequency, expressed as a proportion of the total number of adults (*IF*), and the relative larval viability (*RLV*). A population replacement event is defined as having occurred when the proportion of infected adults stabilizes above or equal to the *MI *value. During each series of simulations, individual parameters were varied singly, while the remaining parameter values were held constant as defined in Table 1. Each parameter was uniformly varied at one one-hundredth intervals from zero to one. At each interval, 1000 simulations were conducted, and the number of successful invasions was recorded to determine the probability of population replacement at that specific parameter value. The uniform sensitivity analysis was implemented for direct comparisons between all parameters across all intervals. Furthermore, previous analyses have not established minimum values for the spread of *Wolbachia*. Additional simulations tested two-way interactions between each of the emphasized parameters by varying two parameters simultaneously and evaluating the probability of population replacement. In the aforementioned simulations, parameters were varied uniformly. One parameter would be held constant while the other parameter varied as described above. The first parameter would then be incremented and the process above would be repeated. The probabilities resulting from two-way interactions were approximately the product of the two parameters and are not discussed further.

## Results

Figure 3 provides an example of the typical population dynamics resulting from model simulations of a *Wolbachia *population replacement event. In the illustrated example, the population begins as cohort of uninfected eggs and stabilizes after approximately 150 days, with variation around a consistent population size and lifestage distribution (Figure 3a). In the example simulation, the introduction of *Wolbachia *occurs at day 800 by introducing blood-fed, gravid adult females at an initial *Wolbachia *infection frequency (*IF*) of 0.5 (Table 1). *IF *is the frequency of *Wolbachia-*infected females relative to the total number of adults such that an *IF *= 1 is synonymous with a 1:1 (infected to uninfected) ratio. Figure 3b illustrates the resulting variation in *Wolbachia *infection frequency in the host population versus time.

Due to the stochastic nature of the model, the number of individuals within each lifestage fluctuates considerably over time (Figure 3a). To examine for temporal patterns in the fluctuations that might correspond to periodic signals such as stage durations or generation time, we performed a spectral analysis on the time series data for both total adult and larval populations via Fast Fourier Transformation [42,43]. The analysis can identify temporal patterns that exist in what appear to be chaotic time series. No pattern was detected by the spectral analysis. Since no period was found, stochasticity appears to be the sole driver of population fluctuations.

Five parameters associated with *Wolbachia *infection were evaluated for their affect on the probability of population replacement. The value of each parameter was varied at one one-hundredth increments, from zero to one, while additional parameters were held constant as defined in Table 1. For each parameter value, the probability of population replacement was determined by the number of successful replacement events occurring in 1000 simulations, for a total of 101,000 simulations per parameter.

**Figure 4:**
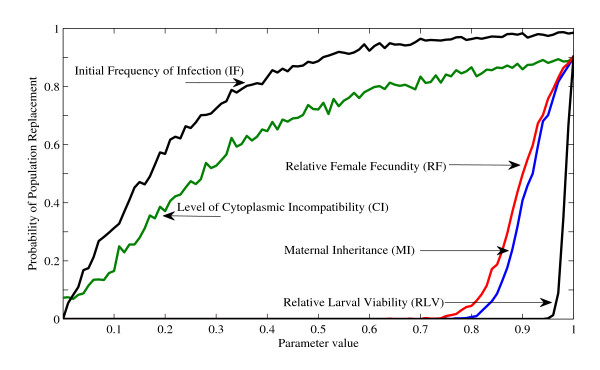
**The probability of population replacement for five *Wolbachia *specific parameters**. *CI *is the level of cytoplasmic incompatibility, *MI *is the level of maternal inheritance, *IF *is the initial frequency of *Wolbachia *infection, *RF *is the relative fecundity of *Wolbachia*-infected adult females, and *RLV *is the relative larval viability. Each line was generated by calculating the probability of a population replacement event at one one-hundredth increments for parameter values between zero and one (n = 1000 simulations/increment). *IF *and *CI *show similar responses to parameter value increases. The probability of population replacement increases, but then asymptotically approaches one. The response curves for *RF*, *MI*, and *RLV *behave similarly, each parameter requiring values to be greater than approximately 0.7. The curves then quickly increase toward one. *RLV *is the most sensitive parameter requiring values approaching 0.95 before a population replacement event can occur. Realistic probabilities of population replacement (i.e., population replacement occurs in greater than 50% of simulations) does not occur until *RLV *is greater than or equal to 0.99.

Maternal inheritance (*MI*), the relative fecundity of adult females (*RF*), and relative larval viability (*RLV*), exhibit strong threshold behavior with population replacement occurring only at parameter values exceeding 0.7 (Figure 4). Specifically, realistic probabilities of population replacement (i.e., > 50% probability of population replacement) require the magnitude of *MI *to be greater than 0.9. Similarly, *RF *must exceed 0.9 before realistic probabilities of population replacement are attained. The probability of population replacement is most sensitive to *RLV*, which requires a value of greater than 0.95 before population replacement can occur. Furthermore, realistic probabilities of population replacement only occur at high *RLV *(≥0.99), despite high maternal inheritance and CI (i.e., all other parameters held at values defined in Table 1).

A different functional relationship is observed with the level of incompatibility (*CI*) and initial *Wolbachia *infection frequency (*IF*), each of which results in response curves that increase asymptotically (Figure 4). Assuming the parameters within Table 1, the model predicts that CI is not necessary for *Wolbachia *to spread (i.e., approximately 7% of simulations resulted in population replacement when *CI *= 0). Realistic probabilities of population replacement occur when *CI *approaches 0.3. Despite perfect CI (i.e., no egg hatch in incompatible crosses), population replacement did not occur in 10% of simulations (Figure 4). Additional simulations confirmed that a 90% probability of population replacement is an absolute maximum given the conditions defined here (Table 1). However, as the magnitude of *IF *increases, the probability of population replacement rapidly approaches one, with realistic probabilities of population replacement occurring when the frequency of infected females approaches 20% (Figure 4).

**Figure 5:**
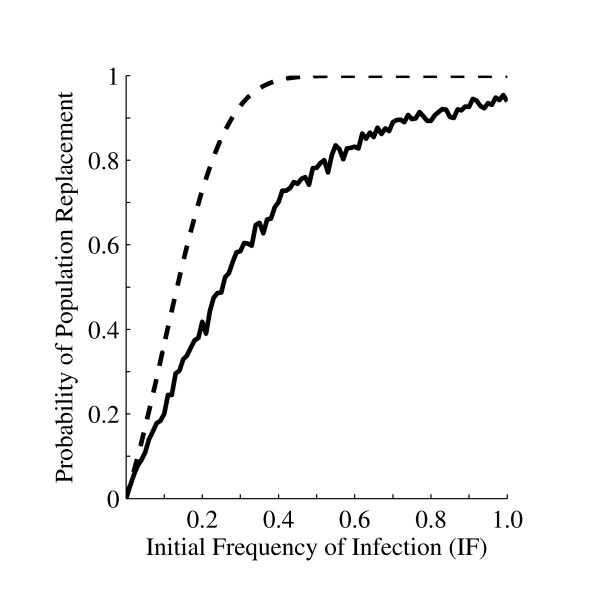
**The probability of population replacement by *Wolbachia *given different initial infection frequencies**. This figure assumes that the relative fecundity of infected females is 0.95, with perfect CI and maternal inheritance. The dashed line indicates the probability of population replacement as calculated by Jansen *et al *[22], and the solid line represents the predictions of this model. Our model predicts lower probabilities at all initial *Wolbachia *infection frequencies, but generates a similar functional response.

The results obtained from the model here were compared to a previously published stochastic model [22]. Table 2 compares the fixation probabilities calculated by the model presented here and those from Jansen *et al. *[22] using the conditions defined in the prior report, which includes the introduction of a single infected female into a population size of 100 and perfect CI. To allow direct comparison, the relative larval viability in our model was set to one. 50,000 simulations were performed for each combination of parameter values used in the prior publication. Both models predict the probability of population replacement decreases when *MI *and *RF *values are less than one (Table 2). Generally, our model predicted lower probabilities of population replacement than the previously published model. However, when either *MI *or *RF *was 80%, the model presented here reported higher probabilities (Table 2). Jansen *et al. *[22] predicted that *Wolbachia *infections with imperfect maternal inheritance and low adult fitness costs (*MI *= *RF *= 0.9) will still invade and establish in a population, but our model predicted no population replacement events (Table 2). The predictions of our model were also compared to those of Jansen *et al. *[22] assuming larger initial frequencies of *Wolbachia *infected individuals (Figure 5). Both models predict an asymptotic increase in the probability of population replacement with increasing magnitude of *IF*, but our model predicts lower probabilities of population replacement (Figure 5).

**Table 2:** The probability of population replacement for given parameter values

*MI*	*RF*		
	
	1.0	0.9	0.8
1.0	0.1023/0.0359	0.0224/0.0089	0.0004/0.0007
0.9	0.0158/0.0060	0.0004/0.0000	/
0.8	0.0001/0.0004	/	/

Jansen *et al. *[22]/model presented here			

The probability of population replacement for given parameter values, assuming perfect CI and the release of a single infected adult female into an uninfected population with a size of 100. The fixation probabilities from our model are generally lower than the values predicted by Jansen *et al*.[22], except when maternal inheritance and the relative fecundity of infected adults is 0.8. The model presented here predicted population replacement would not occur when both maternal inheritance and the relative fecundity of infected adult females were 0.9. All probabilities generated from the model presented here reflect the proportion of population replacement events that occurred per 50,000 simulations of the model. Comparable values were estimated from Figure 2 in Jansen *et al. *[22].

## Discussion

The model presented here examines the probabilities of *Wolbachia *invasion into an isolated uninfected population. The model is unique in its individual-based representation of variation in key traits among adult females and in the resolution of larval dynamics within the host population. The model presented here predicts, as in previous modeling studies, that maternal inheritance (*MI) *and the relative fecundity of adult females (*RF*) are key parameters that determine the potential for population replacement. Specifically, population replacement occurs only at high *MI *or *RF*. In contrast, population replacement can occur at low *CI *or low *IF*. The simulation of adult females as individuals demonstrates that *MI *requires higher parameter values than *RF *for successful population replacement. The new parameter, relative larval viability (*RLV*), like *MI *and *RF*, requires high parameter values before population replacement can occur.

The relative larval viability between *Wolbachia *infected and uninfected individuals (*RLV*) is the most important determinant of population replacement, requiring the highest parameter values for invasion. The model predicts that reductions in infected larval survival can substantially reduce the probability of population replacement (Figure 4). While a majority of prior studies have examined for an effect in adults, recent studies have determined that, at high levels of intraspecific competition, *Wolbachia *infected larvae experience reduced survival [29]. However, few theoretical studies have examined the impact of immature lifestages on the invasion of *Wolbachia*. Here, we demonstrate that reductions in *RLV *will inhibit *Wolbachia *invasion into an uninfected host population.

Recent work has highlighted the prevalence of *Wolbachia*, and its ability to invade populations [1,20]. Studies have suggested that *Wolbachia *infection affects larval survival and development only when intraspecific competition is high [29,44]. Given the predictions from our model, *Wolbachia *can only invade a population when *RLV *is very high. Therefore, the density of conspecifics in larval habitats is predicted to have significant impacts on the probability of population replacement. Similarly, the abundance and variety of larval habitats may have significant impact on the invasion of *Wolbachia*. The distribution, utilization and variety of larval habitats is well known for some insects, particularly mosquitoes [45-48]. Theoretical studies considering the effect of metapopulation structure and larval rearing conditions may elucidate the mechanism by which *Wolbachia *can invade natural populations given low initial infection frequencies.

The level of CI in insects varies widely [44,49-51]. Our model shows that the intensity of CI has relatively little effect on the probability of population replacement when the rate of CI exceeds 60%. Furthermore, when *CI *= 0, the model presented here predicts population replacement can occur at low probabilities (Figure 4). Some *Wolbachia *infections do not cause CI, but are found at high frequencies in natural populations [44,50,52]. Previous theoretical studies indicate that CI or a sex-ratio distorter is not required for population replacement when endosymbionts can alter female traits [44,53]. However, results presented here suggest that non-CI inducing *Wolbachia *infections can establish and persist in a population without increasing or altering host fitness, given high *MI*, *RF*, and *RLV*. Since the population considered by the model presented here is relatively small (N ≈ 110 adults), genetic drift could perhaps influence the probability of population replacement [54]. To investigate the importance of genetic drift, the population size in the model was increased. In model simulations where the total adult population size is greater than approximately 200, population replacement does not occur when there is no effect of CI (i.e. *CI *= 0). However, when population size is increased, the general response patterns in Figure 4 are not altered.

High maternal inheritance rates have been observed consistently in natural populations [55-57]. Furthermore, theoretical studies predict the probability of population replacement declines as maternal inheritance decreases [12,21,22]. Similar to previous studies, results presented here suggest that maternal inheritance (*MI*) must be high for a *Wolbachia *infection to invade an uninfected population and persist. Specifically, *MI *must be higher than 90% to attain a realistic probability of population replacement.

The effect of *Wolbachia *infections on adult female fitness has been well documented empirically and theoretically [11,15,16,22,24,58,59]. Here, as in previous theoretical studies, the model predicts that the relative fecundity of adult females (*RF*) must be high to facilitate population replacement.

For all parameters, the probability of population replacement approached an absolute maximum of 90% given the conditions defined in Table 1. Here, the initially examined *IF *value is relatively high (0.5), analogous to artificial introductions examined in prior theoretical work [25]. Subsequently, lower *IF *values have been simulated (Figure 4), including the introduction of a single, infected female (Table 2). The model predicts that *Wolbachia *invasion can occur at the lowest *IF *values and demonstrates an increasing probability of invasion with the higher introduction levels, with the probability of population replacement approaching 100%. Additional simulations determined that when *IF *is held constant and the total adult population size is increased, the probability of population replacement approaches one given the conditions defined in Table 1. This result suggests genetic drift can affect the probability of population replacement in small populations and may facilitate or hinder the spread of *Wolbachia *from low initial frequencies [54].

The model presented here predicted lower population replacement probabilities than those predicted by previous stochastic models (Table 2 and Figure 5) [22]. Rasgon and Scott [25] noted a similar behavior where implementing population age-structure and overlapping generations increased deterministic thresholds. The inclusion of additional life stages and stage-structure in this stochastic model may explain the reduced probabilities of population replacement. However, the model presented here predicted marginally higher probabilities of population replacement when either maternal inheritance or the relative fecundity of infected females had a magnitude of 0.8. The increased probability of population replacement predicted by the model presented here is likely a result of the individual-based representation of the adult female life stage that includes stochastic survival.

The model here addresses a single, panmictic, isolated population but could be expanded to include metapopulation structure. If introduction events can be assumed to occur randomly, then the surrounding subpopulations should generally tend to inhibit population replacement, because migration between subpopulations would dilute the proportion of infected individuals. However, as demonstrated here, genetic drift may influence the invasion of *Wolbachia *in smaller subpopulations. The spatial spread of *Wolbachia *has been assessed analytically by others and defines the conditions needed for *Wolbachia *to spread through space [20,24].

The majority of models that address the invasion of *Wolbachia *into uninfected populations have examined populations without lifestage subdivisions, suggesting that additional empirical studies focused on understanding larval dynamics are needed [34]. Many of the parameters defined here may be difficult to determine in natural populations [25], but our results demonstrate the importance of understanding the role of life history parameters and their interactions, despite the difficulties. Furthermore, the sensitivity analysis of the model presented here demonstrates that the magnitudes of particular parameters strongly influence the potential for spread and establishment of *Wolbachia*; these (e.g., *Wolbachia *effects on immature fitness) should be the focus of future empirical and theoretical studies. Future theoretical studies could further address parameter sensitivity by hyper-cube sampling, but this would require information about the distribution of parameters to investigated [60].

## Conclusions

*Wolbachia *is currently being utilized as the basis for a gene drive strategy in open field releases of *Aedes aegypti *[61,62]; however, the predictions of the model presented here suggest that minute reductions in *RLV *can inhibit population replacement. Research needs to focus on understanding the effects of novel *Wolbachia *infections on immature lifestages. Xi *et al. *[63] demonstrated that novel *Wolbachia *infections can establish in a new host species and replace an uninfected population, but the initial frequency of *Wolbachia *infected individuals needed to replace the population was higher than predicted. The authors suggested that differences in survival of immature lifestages could explain their results. Results presented here indicate that even reductions in *RLV *that are difficult to detect empirically will substantially reduce the probability of population replacement.

The rapid decline in the probability of population replacement associated with reduced larval viability indicates that empirical studies directed toward quantifying the effects of endosymbionts on immature insects are important for understanding and predicting *Wolbachia *invasion events. Recent empirical studies also suggest that a more complete understanding of the effects of *Wolbachia *on the immature life stages is generally needed through additional empirical and theoretical studies [28-30].

## Authors' contributions

The original model was conceptualized by PRC, JWM and ES in a class taught by PHC. The model was revised by YH, PHC, and SLD. JWM and ES parameterized equations, which were derived by PHC. PRC coded the model and simulations in MATLAB, which was later optimized by PRC, PHC, and YH. The manuscript was written by PRC, PHC, and SLD. All authors have read and approved the final manuscript.

## Supplementary Material

Additional file 1 **Model parameters, detailed equation appendix and sensitivity analysis**. Portable Document File (pdf) containing all parameters, initial parameter values, and equations utilized by the model. Model development is discussed, and includes references from which each equation was developed/parameterized. Also includes the sensitivity analysis of all population dynamic parameters and discussion about the robustness of model predictions Click here for file

## References

[B1] Hilgenboecker K Hammerstein P Schlattmann P Telschow A Werren JH How many species are infected with *Wolbachia*? - A statistical analysis of current data Fems Microbiology Letters 2008 281 2 215 220 10.1111/j.1574-6968.2008.01110.x 18312577 PMC2327208

[B2] Werren JH Biology of *Wolbachia* Annu Rev Entomol 1997 42 587 609 10.1146/annurev.ento.42.1.587 15012323

[B3] Werren JH Baldo L Clark ME *Wolbachia*: Master manipulators of invertebrate biology Nature Reviews Microbiology 2008 6 10 741 751 10.1038/nrmicro1969 18794912

[B4] Hurst GDD Jiggins FM von der Schulenburg JHG Bertrand D West SA Goriacheva II Zakharov IA Werren JH Stouthamer R Majerus MEN Male-killing *Wolbachia *in two species of insect P Roy Soc Lond B Bio 1999 266 1420 735 740 10.1098/rspb.1999.0698

[B5] Hornett EA Charlat S Wedell N Jiggins CD Hurst GDD Rapidly shifting sex ratio across a species range Curr Biol 2009 19 19 1628 1631 10.1016/j.cub.2009.07.071 19747825

[B6] Bouchon D Rigaud T Juchault P Evidence for widespread *Wolbachia *infection in isopod crustaceans: Molecular identification and host feminization P Roy Soc Lond B Bio 1998 265 1401 1081 1090 10.1098/rspb.1998.0402 PMC1689171 9684374

[B7] Kobayashi Y Telschow A Cytoplasmic feminizing elements in a two-population model: Infection dynamics, gene flow modification, and the spread of autosomal suppressors J Evol Biol 2010 23 12 2558 2568 10.1111/j.1420-9101.2010.02116.x 20939837

[B8] Huigens ME Luck RF Klaassen RHG Maas MFPM Timmermans MJTN Stouthamer R Infectious parthenogenesis Nature 2000 405 6783 178 179 10.1038/35012066 10821272

[B9] Kremer N Charif D Henri H Bataille M Prevost G Kraaijeveld K Vavre F A new case of *Wolbachia *dependence in the genus *Asobara*: Evidence for parthenogenesis induction in *Asobara japonica* Heredity 2009 103 3 248 256 10.1038/hdy.2009.63 19513092

[B10] Stouthamer R Russell JE Vavre F Nunney L Intragenomic conflict in populations infected by parthenogenesis inducing *Wolbachia *ends with irreversible loss of sexual reproduction BMC Evol Biol 2010 10 10.1186/1471-2148-10-229 PMC2927591 20667099

[B11] Turelli M Hoffmann AA Cytoplasmic incompatibility in *Drosophila simulans *dynamics and parameter estimates from natural populations Genetics 1995 140 4 1319 1338 7498773 10.1093/genetics/140.4.1319 PMC1206697

[B12] Farkas JZ Hinow P Structured and unstructured continuous models for *Wolbachia *infections Bull Math Biol 2010 72 8 2067 2088 10.1007/s11538-010-9528-1 20232169

[B13] Dobson SL Fox CW Jiggins FM The effect of *Wolbachia*-induced cytoplasmic incompatibility on host population size in natural and manipulated systems P Roy Soc Lond B Bio 2002 269 1490 437 445 10.1098/rspb.2001.1876 PMC1690924 11886634

[B14] Engelstadter J Telschow A Cytoplasmic incompatibility and host population structure Heredity 2009 103 3 196 207 10.1038/hdy.2009.53 19436325

[B15] Caspari E Watson GS On the evolutionary importance of cytoplasmic sterility in mosquitos Evolution 1959 13 4 568 570 10.2307/2406138

[B16] Fine PEM Dynamics of symbiote-dependent cytoplasmic incompatibility in Culicine mosquitos J Invertebr Pathol 1978 31 1 10 18 10.1016/0022-2011(78)90102-7 415090

[B17] Hoffmann AA Turelli M Harshman LG Factors affecting the distribution of cytoplasmic incompatibility in *Drosophila simulans* Genetics 1990 126 4 933 948 2076821 10.1093/genetics/126.4.933 PMC1204290

[B18] Hurst LD The evolution of cytoplasmic incompatibility or when spite can be successful J Theor Biol 1991 148 2 269 277 10.1016/S0022-5193(05)80344-3 2016892

[B19] Turelli M Evolution of incompatibility-inducing microbes and their hosts Evolution 1994 48 5 1500 1513 10.2307/2410244 28568404

[B20] Turelli M Hoffmann AA Rapid spread of an inherited incompatibility factor in California *Drosophila* Nature 1991 353 6343 440 442 10.1038/353440a0 1896086

[B21] Egas M Vala F Breeuwer JAJ On the evolution of cytoplasmic incompatibility in haplodiploid species Evolution 2002 56 6 1101 1109 12144012 10.1111/j.0014-3820.2002.tb01424.x

[B22] Jansen VAA Turelli M Godfray HCJ Stochastic spread of *Wolbachia* Proceedings of the Royal Society B-Biological Sciences 2008 275 1652 2769 2776 10.1098/rspb.2008.0914 PMC2605827 18755670

[B23] Haygood R Turelli M Evolution of incompatibility inducing microbes in subdivided host populations Evolution 2009 63 2 432 447 10.1111/j.1558-5646.2008.00550.x 19154372

[B24] Turelli M Cytoplasmic incompatibility in populations with overlapping generations Evolution 2010 64 1 232 241 10.1111/j.1558-5646.2009.00822.x 19686264

[B25] Rasgon JL Scott TW Impact of population age structure on *Wolbachia *transgene driver efficacy: Ecologically complex factors and release of genetically modified mosquitoes Insect Biochem Molec 2004 34 7 707 713 10.1016/j.ibmb.2004.03.023 15242712

[B26] Dong P Wang JJ Hu F Jia FX Influence of *Wolbachia *infection on the fitness of the stored-product pest *Liposcelis tricolor *(Psocoptera: Liposeelididae) J Econ Entomol 2007 100 4 1476 1481 10.1603/0022-0493(2007)100[1476:IOWIOT]2.0.CO;2 17849905

[B27] Islam MS Dobson SL *Wolbachia *effects on *Aedes albopictus *(Diptera: Culicidae) immature survivorship and development J Med Entomol 2006 43 4 689 695 10.1603/0022-2585(2006)43[689:WEOAAD]2.0.CO;2 16892625

[B28] McMeniman CJ O'Neill SL A virulent *Wolbachia *infection decreases the viability of the Dengue vector *Aedes aegypti *during periods of embryonic quiescence Plos Neglect Trop D 2010 4 7 10.1371/journal.pntd.0000748 PMC2903475 20644622

[B29] Gavotte L Mercer DR Stoeckle JJ Dobson SL Costs and benefits of *Wolbachia *infection in immature *Aedes albopictus *depend upon sex and competition level J Invertebr Pathol 2010 105 3 341 346 10.1016/j.jip.2010.08.005 20807539 PMC3401884

[B30] Gavotte L Mercer DR Vandyke R Mains JW Dobson SL *Wolbachia *infection and resource competition effects on immature *Aedes albopictus *(Diptera: Culicidae) J Med Entomol 2009 46 3 451 459 10.1603/033.046.0306 19496412 PMC2719795

[B31] Barbosa PP Greenough MT N C Overcrowding of mosquito populations: Responses of larva *Aedes aegypti *to stress Environmental Entomology 1972 1 1 89 93

[B32] Peters TM Barbosa P Influence of population-density on size, fecundity, and developmental rate of insects in culture Annu Rev Entomol 1977 22 431 450 10.1146/annurev.en.22.010177.002243

[B33] Dye C Model for the population-dynamics of the Yellow Fever mosquito, *Aedes aegypti* J Anim Ecol 1984 53 1 247 268 10.2307/4355

[B34] Magori K Legros M Puente ME Focks DA Scott TW Lloyd AL Gould F Skeeter Buster: A stochastic, spatially explicit modeling tool for studying *Aedes aegypti *population replacement and population suppression strategies Plos Neglect Trop D 2009 3 9 10.1371/journal.pntd.0000508 PMC2728493 19721700

[B35] Focks DA Haile DG Daniels E Mount GA Dynamic life table model for *Aedes aegypti *(Diptera: Culicidae) - Simulation and validation J Med Entomol 1993 30 6 1018 1028 8271243 10.1093/jmedent/30.6.1018

[B36] Southwood T Murdie G Yasuno M Tonn R Reader P Studies on the life budget of *Aedes aegypti *in Wat Samphaya, Bangkok, Thailand Bulletin of the World Health Organization 1972 46 211 226 4537483 PMC2480713

[B37] Trpis M Hausermann W Dispersal and other population parameters of *Aedes aegypti *in an African village and their possible significance in epidemiology of vector-borne diseases Am J Trop Med Hyg 1986 35 6 1263 1279 3789275 10.4269/ajtmh.1986.35.1263

[B38] Blackmore MS Lord CC The relationship between size and fecundity in *Aedes albopictus* Journal of Vector Ecology 2000 25 2 212 217 11217219

[B39] Lounibos LP Rey JR Frank JH Ecology of mosquitoes: Proceedings of a workshop 1985 Vero Beach, Fla.: Florida Medical Entomology Laboratory

[B40] Gillett JD Roman EA Phillips V Erratic hatching in *Aedes *eggs - New interpretation P Roy Soc Lond B Bio 1977 196 1123 223 232 10.1098/rspb.1977.0038 16266

[B41] Christophers SR Aëdes aegypti (L.), the Yellow fever mosquito; its life history, bionomics, and structure 1960 Cambridge Eng.: University Press

[B42] Wijnen H Naef F Young MW Molecular and statistical tools for circadian transcript profiling Methods Enzymol 2005 393 341 365 15817298 10.1016/S0076-6879(05)93015-2

[B43] Keegan KP Pradhan S Wang JP Allada R Meta-analysis of *Drosophila *circadian microarray studies identifies a novel set of rhythmically expressed genes PLoS Comput Biol 2007 3 11 2087 2110 10.1371/journal.pcbi.0030208 PMC2098839 17983263

[B44] Hoffmann AA Clancy D Duncan J Naturally-occurring *Wolbachia *infection in *Drosophila simulans *that does not cause cytoplasmic incompatibility Heredity 1996 76 1 8 10.1038/hdy.1996.1 8575931

[B45] Aldstadt J Koenraadt CJM Fansiri T Kijchalao U Richardson J Jones JW Scott TW Ecological modeling of *Aedes aegypti *(L.) pupal production in rural Kamphaeng Phet, Thailand Plos Neglect Trop D 2011 5 1 10.1371/journal.pntd.0000940 PMC3022520 21267055

[B46] Harrington LC Ponlawat A Edman JD Scott TW Vermeylen F Influence of container size, location, and time of day on oviposition patterns of the Dengue vector, *Aedes aegypti*, in Thailand Vector-Borne Zoonotic Dis 2008 8 3 415 423 10.1089/vbz.2007.0203 18279006 PMC2978047

[B47] Harrington LC Ponlawat A Scott TW Edman JD Does container size influence oviposition choices of the dengue vector *Aedes aegypti*? Am J Trop Med Hyg 2005 73 6 914

[B48] Koenraadt CJM Aldstadt J Kijchalao U Sithiprasasna R Getis A Jones JW Scott TW Spatial and temporal patterns in pupal and adult production of the Dengue vector *Aedes aegypti *in Kamphaeng Phet, Thailand Am J Trop Med Hyg 2008 79 2 230 238 18689629

[B49] Mercot H Charlat S *Wolbachia *infections in *Drosophila melanogaster *and *D. simulans*: Polymorphism and levels of cytoplasmic incompatibility Genetica 2004 120 1-3 51 59 15088646 10.1023/b:gene.0000017629.31383.8f

[B50] Charlat S Le Chat L Mercot H Characterization of non-cytoplasmic incompatibility inducing *Wolbachia *in two continental African populations of *Drosophila simulans* Heredity 2003 90 1 49 55 10.1038/sj.hdy.6800177 12522425

[B51] Zabalou S Apostolaki A Pattas S Veneti Z Paraskevopoulos C Livadaras I Markakis G Brissac T Mercot H Bourtzis K Multiple rescue factors within a *Wolbachia *strain Genetics 2008 178 4 2145 2160 10.1534/genetics.107.086488 18430940 PMC2323804

[B52] Hoffmann AA Hercus M Dagher H Population dynamics of the *Wolbachia *infection causing cytoplasmic incompatibility in *Drosophila melanogaster* Genetics 1998 148 1 221 231 9475734 10.1093/genetics/148.1.221 PMC1459765

[B53] Hayashi TI Marshall JL Gavrilets S The dynamics of sexual conflict over mating rate with endosymbiont infection that affects reproductive phenotypes J Evol Biol 2007 20 6 2154 2164 10.1111/j.1420-9101.2007.01429.x 17887971

[B54] Hedrick PW Genetics of populations 2011 4 Sudbury, Mass.: Jones and Bartlett Publishers

[B55] Narita S Nomura M Kageyama D Naturally occurring single and double infection with *Wolbachia *strains in the butterfly *Eurema hecabe*: transmission efficiencies and population density dynamics of each *Wolbachia *strain Fems Microbiology Ecology 2007 61 2 235 245 10.1111/j.1574-6941.2007.00333.x 17506822

[B56] Poinsot D Montchamp-Moreau C Mercot H *Wolbachia *segregation rate in *Drosophila simulans *naturally bi-infected cytoplasmic lineages Heredity 2000 85 2 191 198 10.1046/j.1365-2540.2000.00736.x 11012722

[B57] Rasgon JL Scott TW *Wolbachia *and cytoplasmic incompatibility in the California *Culex pipiens *mosquito species complex: Parameter estimates and infection dynamics in natural populations Genetics 2003 165 4 2029 2038 14704183 10.1093/genetics/165.4.2029 PMC1462871

[B58] Weeks AR Reynolds KT Hoffmann AA Mann H *Wolbachia *dynamics and host effects: What has (and has not) been demonstrated? Trends Ecol Evol 2002 17 6 257 262 10.1016/S0169-5347(02)02480-1

[B59] Weeks AR Turelli M Harcombe WR Reynolds KT Hoffmann AA From parasite to mutualist: Rapid evolution of *Wolbachia *in natural populations of *Drosophila* PLoS Biol 2007 5 5 997 1005 10.1371/journal.pbio.0050114 PMC1852586 17439303

[B60] Kiparissides A Kucherenko SS Mantalaris A Pistikopoulos EN Global sensitivity analysis challenges in biological systems modeling Ind Eng Chem Res 2009 48 15 7168 7180 10.1021/ie900139x

[B61] Marshall JM The effect of gene drive on containment of transgenic mosquitoes J Theor Biol 2009 258 2 250 265 10.1016/j.jtbi.2009.01.031 19490857

[B62] Enserink M Australia to test 'mosquito vaccine' against human disease Science 2010 330 6010 1460 1461 10.1126/science.330.6010.1460 21148356

[B63] Xi ZY Khoo CCH Dobson SL *Wolbachia *establishment and invasion in an *Aedes aegypti *laboratory population Science 2005 310 5746 326 328 10.1126/science.1117607 16224027

