# Supplementary material for: Wolbachia infections that reduce immature insect survival: Predicted impacts on population replacement
Source: BMC Evol Biol. 2011 Oct 5;11:290. doi: 10.1186/1471-2148-11-290 (PMC3202246; doi:10.1186/1471-2148-11-290)
Supplement: Additional file 1 — Model parameters, detailed equation appendix and sensitivity analysis. Portable Document File (pdf) containing all parameters, initial parameter values, and equations utilized by the model. Model development is discussed, and includes references from which each equation was developed/parameterized. Also includes the sensitivity analysis of all population dynamic parameters and discussion about the robustness of model predictions [file 1471-2148-11-290-S1.PDF]

Electronic Supplementary Material

***Wolbachia* infections that reduce immature insect survival: Predicted impacts on population replacement**

Model Parameters

symbol	definition	initial value	reference
*CI**	level of CI in incompatible crosses (proportion not hatching in incompatible crosses)	0.999	-
*MI**	level of maternal inheritance (proportion of offspring receiving infection)	0.999	-
*RF**	relative fecundity of infected females to uninfected females	0.999	-
*RLV**	relative larval viability of infected larvae to uninfected larvae	0.999	-
*IF**	introduction ratio of gravid infected females to the total adult population at the time of introduction	0.500	-
s	number of larval developmental stages	30	-
u	egg production rate	10.486	derived from equations by Lounibos *et al*. [1] and Blackmore and Lord [2]
v	female mass coefficient	3.317	derived from equations by Lounibos *et al*. [1] and Blackmore and Lord [2]
w	female mass intercept	0.017	derived from equations by Lounibos *et al*. [1] and Blackmore and Lord [2]
z	female mass exponent	0.333	derived from equations by Lounibos *et al*. [1] and Blackmore and Lord [2]
m_x	theoretical mass of larva at time *T*	0.005	calculated from laboratory experiment

symbol	definition	initial value	reference
T_{f0}	female development time at which mass at pupation is $m_x/2$ days	10.480	calculated from data presented in Gavotte *et al*. [3]
T_{m0}	male development time at which mass at pupation is $m_x/2$ days	8.075	calculated from data presented in Gavotte *et al.* [3]
μ	baseline larval mortality (i.e. mortality in the absence of age and density dependent effects)	0.100	calculated from data presented in Gavotte *et al*. [3]
α	density dependent mortality coefficient	0.025	-
β	density dependent mortality exponent	0.061	-
γ	stage-dependent mortality coefficient	1.150	-
ε	stage-dependent mortality exponent	1.200	-
Δt	time step	1.000	-
g	per capita mortality rate of adult females	0.106	Trpis and Hausermann [4]
j_m	maximum larval male development rate	6.310	calculated from data presented in Gavotte *et al*. [3]
j_f	maximum larval female development rate	5.469	calculated from data presented in Gavotte *et al*. [3]
h_m	asymptotic minimum development rate	1.500	calculated from data presented in Gavotte *et al*. [3]
h_f	asymptotic minimum development rate	1.100	calculated from data presented in Gavotte *et al*. [3]
q	density-dependent development coefficient	0.035	-
k_m	male growth coefficient	0.153	-
k_f	female growth coefficient	0.174	-
S_E	survival of individuals in the egg stage	1	-
S_P	survival of individuals in the pupa stage	1	-

symbol	definition	initial value	reference
S_M	survival of individuals in the adult male stage (both infected and uninfected)	0.600	Trpis and Hausermann [4]
H_3	proportion of eggs hatching early	0.200	Gillet *et al.* [5]
P_B	probability a female mosquito dies before bloodfeeding on host	0.100	Trpis and Hausermann [4]
P_A	probability a female mosquito dies after bloodfeeding on host	0.100	Trpis and Hausermann [4]
P_C	probability female changes hosts during bloodfeeding	0.050	Trpis and Hausermann [4]

Table S1. Glossary of notation, including the initial values for each parameter. In all subsequent model runs, each value remains constant while one key parameter is varied. Key parameters are identified by an asterisk after its symbol.

Extended Appendix

Although this model can be generalized to many holometabolous insect systems, our particular case study involved mosquitoes in the genus *Aedes*. The key functions were specifically designed to address mosquito population dynamics.

$$R = \frac{(j-h)\Delta t e^{-qB} + h\Delta t}{s} \tag{1}$$

The development rate R (developmental stage units) is an exponential function of time-step duration (Δt) and total larval population mass B (units of mass), with an asymptotic minimum that reflects the minimum development rate that larva will develop when larval competition is

high. j is the maximum development rate (developmental stage units), h is the asymptotic minimum development rate (developmental stage units), Δt is the time step (units of time), q is the density-dependent development coefficient (units of $(\text{mass})^{-1}$), and s is the total number of developmental stages. The rate of development increases inversely with larval competition, and the rate is dependent on the overall number of divisions in the larval stage. This function was developed to describe patterns in experimental data presented by Gavotte *et al*. [1]. Default parameter values were derived from curve fitting raw data. Furthermore, a similar functional relationship between immature development time and immature density has been described previously [6-7].

$$S_L = e^{-(\mu + \alpha B^{\beta} + \gamma d^{-\varepsilon})\Delta t} \quad (2)$$

Larval survival, S_L, is an exponential function where the form of the function is governed by the development stage and density of the larvae. μ is the baseline mortality rate of mosquito larvae in the absence of competition (units of $(\text{time})^{-1}$). α is the coefficient controlling density dependent mortality (units of $(\text{time})^{-1}$). B is the total larval biomass (dimensionless), β is the exponent controlling density dependent mortality (dimensionless), γ is the coefficient that decreases mortality as development stage increases (units of $(\text{time})^{-1}$), d is the developmental stage index, ε is the exponent that decreases mortality as development stage increases (dimensionless), and Δt which is the time step (units of time) thus, S_L increases as the stage of larval development increases but will decrease when competition increases. The equation presented here was modified from an equation presented by Dye [8] to incorporate stage dependent mortality and is

similar to other simulation models [9-10]. Furthermore, the survival from eggs to pupae was similar to that described by Southwood *et al*. [11].

$$M = \frac{m_x e^{k(d-1)}}{1 + \frac{1-c}{c}^{\frac{T_{0-T}}{T_0}}} \quad (3)$$

This function depicts a reverse sigmoid relationship for mosquito body mass, M (units of mass), versus development time. The assumption here is development time is lengthened due to higher levels of intraspecific competition. m_x (units of mass) is the theoretical maximum mass of a given mosquito at time T. m_x is linked to c (dimensionless), which is the percent of m_x that is attainable. k (dimensionless) is the growth coefficient; T_0 (dimensionless) is the development time at which mass at pupation is $m_x/2$ days, and T (dimensionless) is development time. d (dimensionless) represents the total number of development stages completed by the larval cohort. The values c and m_x were derived from measurements taken from first instar larva. A cohort of larvae were dried, and weighed, and the average was taken. The value of c was arbitrarily set to 0.999 and m_x was then back calculated. The equation and parameter values presented here were derived to fit data published by Gavotte *et al*. [3].

$$F_s = e^{-gA} \quad (4)$$

Female survivorship, F_s, is an exponential decline where the survivorship probability decreases when the age of the female increases. g is the per capita mortality rate of adult females (units of

$(time)^{-1}$) and *A* is the current age of the female (units of time). In general, over time, the probability that a female will die increases with the age of the female. The equation here is taken directly from Trpis and Hausermann [4].

$$E = u\Delta t e^{v(M_f + w)^z} \tag{5}$$

Overall egg production, *E*, is governed by body mass, which is redefined from Lounibos [1] to mean body mass (which Blackmore and Lord relates to wing length in their equation). *u* is the egg production rate (units of $(time)^{-1}$); Δt is the time step (units of time); *v* is the female mass coefficient (units of $(mass)^{-1}$); M_f is the body mass of the ovipositing female (units of mass); *w* is the female mass intercept (units of mass), and *z* is the female mass exponent (dimensionless). Female egg production is a composite equation derived by combining two equations that were published previously in Blackmore and Lord [2] and Lounibos [1].

Sensitivity analysis

The model presented here was designed to (1) represent the life history of holometabolous insects and (2) clarify how changes to life history and *Wolbachia*-related parameters affect the probability of population replacement by *Wolbachia*. To assess the robustness of this model, a detailed sensitivity analysis was conducted for each parameter defined in Table 1 (excluding *s* and Δt). *s* (stage index) can be set to any arbitrary value. The number of stages chosen should reflect the desired detail in larval population dynamics. If larval dynamics is likely not an important factor in overall population dynamics, then the number of

stages could be small (approximately the true number of larval instars or less). When larval temporal dynamics and survival are important, more resolution in the larval stage (i.e. many stages per true larval instar) might provide more accurate results. For each parameter, a measure of sensitivity (MOS) was determined by calculating a ratio of the percent change in the probability of population replacement by *Wolbachia* relative to a ten percent change in the initial parameter value. Parameters were then categorized based on their level of sensitivity into four levels: parameters insensitive to change (MOS ≤ 0.2), parameters moderately sensitive to change (0.2 > MOS ≥ 0.5), parameters strongly sensitive to change (0.5 > MOS ≥ 1.0), and parameters supraproportionally sensitive to change (MOS > 1.0). The probability of population replacement was determined by averaging the number of population replacement events occurring in one hundred simulations per replicate for ten replications.

Table S2 lists each parameter and the corresponding measure of sensitivity. Eight of 28 parameters are demonstrated to be insensitive to change to initial parameter values. These parameters are found in equations dictating development rate (h_m and h_f), oviposition (z), larval survival (β), and mosquito body mass (T_{m0}, and T_{f0}). Also the deterministic male survivorship rate (S_M) and the probability that a female changes host during a bloodmeal (P_C) had no effect on the probability of population replacement predicted by the model.

Nine parameters are shown to be moderately sensitive to changes in the initial parameter value. Of these nine parameters, two affect larval survival (α and μ), two are associated with mortality for bloodfeeding females (P_B and P_A), two determine mosquito body mass (m_x and k_m), and the others affect oviposition, development rate, and the proportion of eggs hatching on day three (w, q, and H_3 respectively).

The remaining eleven parameters altered the probability of population replacement substantially, reaching MOS values exceeding 0.5 and 1.0. Parameters significant to change (0.5 > MOS ≥ 1.0) included the maximum male development rate j_m, the egg production rate u, the per capita rate of female mortality g, and the stage-dependent exponent ε. Six parameters are supraproportionally sensitive to changes in initial parameter values. These parameters affect female development rate (j_f) and body mass (k_f), oviposition (v), and mosquito survival (S_E, S_P and γ).

The model presented here tracks the invasion and establishment of a *Wolbachia* infection in a population size of approximately 110 adult mosquitoes. Parameters that significantly affect the probability of population replacement by *Wolbachia* also tend to decrease the overall population size at which the model reaches equilibrium. With some parameters, a ten percent decrease in the initial parameter value lowers the adult population size considerably (e.g. when $S_E = 0.9$, the average population size during a simulation of the model decreased to less than 50 individuals). In these scenarios, the initial *Wolbachia* infection frequency (*IF*, Table 1) remains the same, but the absolute number of total female adults released decreases with the total adult population size. The model incorporates stochastic adult female survival dictated by the female survivorship function (Equation 4). Adult females that have matured sufficiently to take bloodmeals and oviposit have a survivorship of approximately 65%. Given very small population sizes and stochastic survivorship, *Wolbachia* infections can fail to invade due to stochastic female death (demographic stochasticity).

Parameters that can decrease adult population size occur in all of the equations defined here; for example, k_f and k_m can increase or decrease the biomass of larvae. Increasing larval biomass results in decreased larval survival (*B*, Equation 2) and adult population size.

Furthermore, decreases in k_f can decrease the total number of eggs produced by females since body mass is positively correlated with egg production (M_f, Equation 5). Because mosquito size reflects development time, changes to the maximum development rate, *j*, was sensitive to change. Short mosquito development times reflects ideal rearing conditions (i.e. low larval densities), which increase body mass (*T*, Equation 4). As discussed, increases or decreases in body mass substantially affect total population size in all lifestages. Increasing or decreasing various parameters in the larval survival equation can have a variety of effects on the population size, but decreasing survival reduces population size and population replacement in the example shown (Table S2). Similarly, changing parameters that resulted in lower fecundity (Equation 5) tended to inhibit invasion by *Wolbachia*. Increasing the per capita mortality rate for adult females (*g*, Equation 4) decreases the number of females that survive to produce eggs, decreasing population size and population replacement.

The conclusions from the sensitivity analysis were similar to conclusions described in the main manuscript. In general, the survival of immature lifestages influences the adult population size. As shown above, the number of adults can affect the invasion and establishment of *Wolbachia* by genetic drift when the population size is small. Because these data are typically unavailable, the sensitivity of the model to these parameters underscores the importance of additional empirical work. Also parameters affecting adult female individuals (particularly those which ultimately affect fecundity) are very important to the model's quantitative and qualitative behavior. We therefore emphasize that studies examining the spread of *Wolbachia* should characterize host population dynamics as well as possible, since these features so strongly influence the invasion and establishment of *Wolbachia*.

parameter	measure of sensitivity
S_M	0.03
z	0.06
P_C	0.09
h_f	0.10
β	0.12
T_{m0}	0.13
T_{f0}	0.15
h_m	0.16
m_x	0.21
H_3	0.21
w	0.24
P_B	0.34
α	0.35
P_A	0.36
μ	0.40
q	0.42
k_m	0.46
c	0.54
j_m	0.58
u	0.67
g	0.72
ε	0.85
S_P	1.16
γ	1.44
j_f	1.52
v	2.56
k_f	4.03
S_E	4.72

Table S2. The measure of sensitivity (MOS) for all parameters defined in Table 1. MOS was calculated by increasing or decreasing each parameter by 10% then calculating the rate of change in the probability of population replacement. The parameters were categorized based on the magnitude of their MOS, ranging from insensitive to change (MOS ≤ 0.2), moderately sensitive

to change (0.2 > MOS ≥ 0.5), strongly sensitive to change (0.5 > MOS ≥ 1.0), and supraproportionally sensitive to change (MOS > 1.0).

Works Cited

1. Lounibos LP, Rey JR, Frank JH: **Ecology of mosquitoes : Proceedings of a workshop**. Vero Beach, Fla.: Florida Medical Entomology Laboratory; 1985.
2. Blackmore MS, Lord CC: **The relationship between size and fecundity in *Aedes albopictus***. *Journal of Vector Ecology* 2000, **25**(2):212-217.
3. Gavotte L, Mercer DR, Vandyke R, Mains JW, Dobson SL: ***Wolbachia* infection and resource competition effects on immature *Aedes albopictus* (Diptera: Culicidae)**. *J Med Entomol* 2009, **46**(3):451-459.
4. Trpis M, Hausermann W: **Dispersal and other population parameters of *Aedes aegypti* in an African village and their possible significance in epidemiology of vector-borne diseases**. *Am J Trop Med Hyg* 1986, **35**(6):1263-1279.
5. Gillett JD, Roman EA, Phillips V: **Erratic hatching in *Aedes* eggs - New interpretation**. *P Roy Soc Lond B Bio* 1977, **196**(1123):223-232.
6. Barbosa PP, Peters TM, Greenough NC: **Overcrowding of mosquito populations: Responses of larva *Aedes aegypti* to stress**. *Environmental Entomology* 1972, **1:**89-93.
7. Peters TM, Barbosa P: **Influence of population-density on size, fecundity, and developmental rate of insects in culture**. *Annu. Rev. Entomol.* 1977, **22:**431-450.
8. Dye C: **Models for the population-dynamics of the yellow-fever mosquito, *Aedes-aegypti***. *J. Anim. Ecol.* 1984, **53**247-268.

9. Focks DA, Haile DG, Daniels E, Mount GA: **Dynamic life table model for *Aedes-aegypti* (Diptera, Culicidae) - simulation and validation.** *J Med Entomol* 1993, **30:**1018-1028.

10. Magori K, Legros M, Puente ME, Focks DA, Scott TW, Lloyd AL, Gould F. **Skeeter buster: A stochastic, spatially explicit modeling tool for studying *Aedes aegypti* population replacement and population suppression strategies**. *Plos Neglect Trop D* 2009, **3**.

11. Southwood T, Murdie G, Yasuno M, Tonn R, Reader P: **Studies on the life budget of *Aedes aegypti* in Wat Samphaya, Bangkok, Thailand**. *Bulletin of the World Health Organization* 1972, **46:**211-226.
